# Patterns and predictors of adherence to colorectal cancer screening recommendations in Alberta’s Tomorrow Project participants stratified by risk

**DOI:** 10.1186/s12889-018-5095-4

**Published:** 2018-01-25

**Authors:** Nathan M. Solbak, Jian-Yi Xu, Jennifer E. Vena, Ala Al Rajabi, Sanaz Vaseghi, Heather K. Whelan, S Elizabeth McGregor

**Affiliations:** 1 Cancer Measurement, Outcomes, Research and Evaluation, CancerControl Alberta, Alberta Health Services, Calgary, AB Canada; 2 Department of Community Health and Epidemiology, College of Medicine, University of Saskatchewan, Saskatoon, SK Canada; 3 Department of Health and Physical Education, Faculty of Health, Community and Education, Mount Royal University, Calgary, AB Canada; 4 Population, Public and Indigenous Health, Alberta Health Services, Calgary, AB Canada; 5 Alberta’s Tomorrow Project, CancerControl Alberta, Alberta Health Services, 1820 Richmond Road SW, Calgary, AB T2T 5C7 Canada

**Keywords:** Colorectal cancer, Screening, Colonoscopy, Early diagnosis, Cohort

## Abstract

**Background:**

Colorectal cancer (CRC) screening is an important modifiable behaviour for cancer control. Regular screening, following recommendations for the type, timing and frequency based on personal CRC risk, contributes to earlier detection and increases likelihood of successful treatment.

**Methods:**

To determine adherence to screening recommendations in a large provincial cohort of adults, participants in Alberta’s Tomorrow Project (*n* = 9641) were stratified based on increasing level of CRC risk: age (Age-only), family history of CRC (FamilyHx), personal history of bowel conditions (PersonalHx), or both (Family/PersonalHx) using self-reported information from questionnaires. Provincial and national guidelines for timing and frequency of screening tests were used to determine if participants were up-to-date based on their CRC risk. Screening status was compared between enrollment (2000–2006) and follow-up (2008) to determine screening pattern over time.

**Results:**

The majority of participants (77%) fell into the average risk Age-only strata. Only a third of this strata were up-to-date for screening at baseline, but the proportion increased across the higher risk strata, with > 90% of the highest risk Family/PersonalHx strata up-to-date at baseline. There was also a lower proportion (< 25%) of the Age-only group who were regular screeners over time compared to the higher risk strata, though age, higher income and uptake of other screening tests (e.g. mammography) were associated with a greater likelihood of regular screening in multinomial logistic regression.

**Conclusions:**

The low (< 50%) adherence to regular CRC screening in average and moderate risk strata highlights the need to further explore barriers to uptake of screening across different risk profiles.

## Background

In Canada, an estimated 1 in 13 men and 1 in 16 women will develop colorectal cancer (CRC) in their lifetime [1]. As the third most common cancer [1], there is a need to understand how to best identify individuals at risk and provide appropriate screening recommendations, balancing the goals of public health (lowering incidence and mortality of CRC) with economics (efficient use of the health-care system). The premise of screening is to identify early stage cancer development at a time when preventive measures and treatments are most effective and before progression or metastasis occurs [2]. To date, structured CRC screening programs exist or are proposed in ten Canadian provinces and one territory [3].

CRC screening can be done by home stool testing [fecal occult blood test (FOBT) or fecal immunochemical test (FIT)] or endoscopy (flexible sigmoidoscopy or colonoscopy). Consistent adherence to FOBT has been shown to reduce CRC incidence and mortality by 17% [4] and 11% [5], respectively; FIT is a more recent fecal test with improved patient acceptance and diagnostic accuracy [6]. Reductions in CRC incidence and mortality observed for flexible sigmoidoscopy are similar to the FOBT [7]. Colonoscopy is widely considered the gold standard for CRC screening based on its ability to both visualize and remove polyps and neoplastic lesions in all regions of the colon [8]; although there is only indirect evidence of its efficacy as a screening modality [9]. Observational and modeling studies [10, 11] have suggested a reduction in CRC incidence (67 and 81%) and mortality (65 and 83%) with colonoscopy.

Guidelines recommending colorectal cancer screening were first released in Canada in 2001 (Canadian Task Force on Preventive Health Care (CTFPHC) [12]) and in Alberta in 2008 (Alberta Toward Optimized Practice Clinical Practice Guidelines (provincial guidelines) [13]). Both these guidelines and subsequent updates [14, 15] provide risk-dependent screening recommendations for average, moderate and high-risk individuals. Three main criteria determine the level of risk for CRC and, consequently, the recommended screening method, timing and frequency: age, family history of CRC and personal history of certain bowel conditions [13]. In general, FOBT is recommended for average-risk individuals, whereas endoscopy is recommended for high-risk groups, though the 2008 provincial guideline recommended all three options for average risk screening. Over 90% of CRC cases occur in individuals over 50 years [16], and therefore individuals 50 to 74 years without any other risk factors are considered to be at average-risk. Despite its enhanced ability to detect abnormalities, colonoscopy is not recommended as an initial screening modality for average-risk individuals, due to poor compliance, feasibility and cost concerns [12], but rather as a follow-up to a positive FOBT [13]. Family history of CRC is considered a moderate risk - an individual with a first-degree relative (FDR) with CRC has almost a two-fold increased risk for developing CRC, increasing to almost four-fold with two or more affected relatives [17]. Individuals with certain bowel conditions, including inflammatory bowel diseases (IBD, which includes ulcerative colitis and Crohn’s disease [18]) and/or a history of polyps [19] are considered high-risk. Cumulatively, the risk of CRC is the highest for individuals with both a family history of CRC and an inflammatory bowel condition, with relative risks approaching ten-fold [20]. As the level of risk increases based on age, family and personal medical history, the recommended test, age to commence screening, and optimal screening frequency vary (Table 1).

**Table 1:** Colorectal cancer (CRC) screening guidelines and risk criteria^a^

Risk category	Criteria	Age to commence screening	Screening Test	Screening frequency (years)
Average-risk (Age-only)	Age 50–74 years	50	FOBT	2
			Sigmoidoscopy	5
			Colonoscopy	10
Moderate-risk (FamilyHx)	Family history of CRC in 1 first-degree relative ≥60 years	40	FOBT	2
			Sigmoidoscopy	5
			Colonoscopy	10
	Family history of CRC in 1 first-degree relative < 60 years	40^b^	Colonoscopy	5
	Family history of CRC in 2 or more relatives			
High-risk (PersonalHx)	Personal history of a bowel condition^c^	40^d^	Colonoscopy	5
Highest-risk (Family/PersonalHx)	Personal history of a bowel condition and family history of CRC			

^a^According to the 2008 Alberta Toward Optimized Practice Clinical Practice Guidelines for CRC screening and the 2001 Canadian Task Force on Preventive Health Care

^b^According to the guidelines, colonoscopy could also commence 10 years earlier than the age of the first family member diagnosed with CRC; however, the number of participants that should start screening before 40 (i.e. familial case diagnosed before age 50) was low (*n* = 244) and therefore this criteria was not used in the present study

^c^Bowel condition includes inflammatory bowel diseases (IBD, which includes ulcerative colitis and Crohn’s disease) and/or a history of polyps

^d^The guidelines recommend colonoscopy screening starts at 8–10 years after disease onset; however, age of diagnosis of bowel conditions was not captured in the questionnaires completed by participants and therefore this criteria was not used in the present study

Stratum Descriptions:

Age-only - participants who should commence screening due to age (50–74 years); considered average-risk

FamilyHx - participants with a first-degree relative who has been diagnosed with CRC; considered moderate-risk

PersonalHx - participants with a personal history of a bowel condition or polyps; considered high-risk

Family/PersonalHx - participants with first-degree relative diagnosed with CRC and personal history of a bowel condition or polyps; considered highest-risk

A key component of the guidelines is that the right screening test is used in the right patients; this ensures appropriate patient care along with reduced burden and costs on the health-care system. We have previously reported that when age (50–74 years) was the only indication for testing, the majority of Alberta’s Tomorrow Project (ATP) participants were not up-to-date with CRC screening according to CTFPHC guidelines [21]. However, endoscopy was likely to be more recent than FOBT for participants at moderate and high risk for CRC, suggesting appropriate application and utilization of screening recommendations in higher risk groups. Knowledge of screening status at any one time is important, but consistent screening over time is needed to reduce cancer incidence and mortality. For example, FOBT effectiveness as a screening tool is reduced when patients do not adhere to a regular interval of testing [22]. Predictors of repeated mammography [23] and prostate [24] screenings have been investigated, however, available data regarding predictors of repeated CRC screenings are limited [25, 26]. Here, we expand on previous cross-sectional analyses by our group [21] to determine screening behaviour of ATP participants based on stratified CRC risk, determine screening status and patterns and reasons for screening, as well as to identify predictors for regular screening behaviour.

## Methods

### Cohort design and data collection

ATP is a prospective cohort of ~ 55,000 Albertans established in 2000 to study the etiology of cancer and chronic diseases. A full description of study feasibility, design and enrollment are described elsewhere [27, 28]. Briefly, Albertans aged 35–69 years, with no history of cancer except non-melanoma skin cancer, were recruited throughout the province. At enrollment, participants completed the Health and Lifestyle Questionnaire (HLQ; completed between 2000 and 2008). The HLQ collected information on personal and family health history and cancer screening behaviours, as well as reproductive history, smoking habits, anthropometric variables and sociodemographic characteristics. In 2008, a follow-up questionnaire (Survey 2008) was administered; this questionnaire was designed to collect updated information on personal and family health history and screening tests. Ethics was granted by the Health Research Board of Alberta – Cancer Committee (Ethics ID: 25,985). Written consent was collected from each participant at enrollment into the study.

Inclusion in the current study was restricted to individuals aged 50–69 years at enrollment, unless a family history of CRC and/or a personal history of a bowel condition (including polyps) was reported at HLQ; in these individuals, inclusion was extended to age 40–69 years since these individuals should commence screening at an earlier age as suggested by provincial guidelines [13]. Participants who were recruited as the second individual from the same household (*n* = 230), diagnosed with cancer prior to 2008 (*n* = 491), pregnant at enrollment or follow-up (*n* = 69), and participants who completed the HLQ and Survey 2008 with less than 2 years between completions (*n* = 1922) were excluded from this analysis. The exclusion based on years between completions was done to reduce the possibility that the same screening test was reported at enrollment and follow-up. In addition, provincial guidelines do not provide recommendations for individuals ≥75 years and suggest screening may continue but need to be considered along with other health indications and estimated life expectancy; therefore, individuals age ≥ 75 years at follow-up were also excluded (*n* = 141). The average length of time between completion of enrollment and follow-up surveys was mean (SD) = 4.2(2.1) years. The final sample size was *n* = 9641 adults.

### Assessment of screening behaviours

Based on the provincial [13] and CTFPHC guidelines [12] in place at the time of data collection from the cohort, and using information collected at enrollment, participants were allocated into one of four mutually exclusive strata by increasing level of risk: 1) average-risk participants based on age (Age-only; 50–74 years), 2) moderate-risk participants who reported a family history of CRC (FamilyHx) in a first-degree relative, 3) high-risk participants with a personal bowel condition (PersonalHx; chronic inflammatory bowel disease or history of polyps), or 4) highest-risk participants who indicated both a family history of CRC and a personal history of a bowel condition (Family/PersonalHx). The primary screening tests available to Albertans over the time course of data collection were FOBT and endoscopy [13].

Participants’ screening status at enrollment and follow-up was categorized as:

- “up-to-date” if they reported their last screening test (FOBT, sigmoidoscopy, or colonoscopy) within the timeframe recommended by the guidelines
- “not-up-to-date” if their last reported screening test fell outside the recommended timeframe, and,
- “never” if they reported they had never had a screening test.

Timeframes recommended by the guidelines and used here for categorization are specific to stratum risk and screening test (Table 1). Colonoscopy and sigmoidoscopy were asked as one question at enrollment (i.e. combined endoscopy screening question), but separated into individual questions at follow-up. To allow for a direct comparison between enrollment and follow-up, the colonoscopy and sigmoidoscopy questions at follow-up were combined to create an endoscopy screening status variable. In addition, since the endoscopy types were combined into one question at enrollment (and therefore it was not possible to differentiate between sigmoidoscopy and colonoscopy), a 5 year cut-off was applied as the recommended screening timeframe for endoscopy at enrollment for all participants. All other test-specific cut-offs matched the timeframes recommended by the guidelines (Table 1). For the purpose of this study, “overall CRC screening” status was defined at each timepoint (enrollment and follow-up) based on status for *either* FOBT or endoscopy according to the following priority: up-to-date > not-up-to-date > never (e.g. if a participant was not-up-to-date for FOBT but was up-to-date for endoscopy, they would be considered up-to-date for overall CRC screening).

Using the determined screening status at enrollment and follow-up, we derived four “patterns” of screening behaviours, individually for FOBT, endoscopy and overall CRC screening:

- regular screener - participants who were “up-to-date” at both enrollment and follow-up
- new screener - participants who were “never” or “not-up-to-date” at enrollment, but were “up-to-date” at follow-up
- episodic screener - participants who were “not-up-to-date” or “up-to-date” at enrollment, but “not up-to-date” at follow-up; and,
- non-screener - participants who indicated “never” at both enrollment and follow-up.

Participants categorized as “up-to-date” or “not-up-to-date” for a CRC screening type at enrollment and “never” at follow-up were excluded from both screening status and pattern analyses due to inconsistencies in reporting (*n* = 773 FOBT, *n* = 206 endoscopy, *n* = 509 overall CRC screening). There were no participants who reported “never” at enrollment and “not-up-to-date” at follow-up. In addition, the current analysis focused only on CRC screening tests; however, information for other cancer screening tests (mammography and prostate-specific antigen; PSA) were available from enrollment and follow-up questionnaires and past history of these tests were used as predictor variables.

### Reasons for screening at follow-up

On the follow-up questionnaire, participants who indicated that they had received a screening test were also asked to choose a reason for having the test. The list of reasons included: age; part of regular checkup/routine screening; family history of CRC; signs or symptoms of a possible problem; follow-up of previous problem; or other (open text field where participants could write an alternate answer). The open text category comprised < 1% of responses and thus was excluded from analysis. Answers were allocated into three categories according to a priority hierarchy (from highest to lowest), associated with the CRC guidelines [12, 13]: physical problem (signs of a possible problem or follow-up of previous problem), family history of CRC, and regular checkup or age. Participants were instructed to “choose all that apply”, but for this analysis participants were assigned to only one category based on the highest priority reason given (e.g. if a participant chose both family history and regular checkup, they were assigned to the family history category).

### Statistical analysis

Sociodemographic characteristics of participants are presented as mean (standard deviation, SD) for age, and count (percent) for categorical variables. Multinomial logistic regression models were used to assess the association between CRC screening patterns and potential predictors. Regular screeners were assigned as the reference level for screening pattern analyses. The estimated associations are presented as odds ratios (ORs) and 95% confidence intervals (CIs). All estimations were adjusted for age (continuous), body mass index (BMI; continuous), residential area (rural/urban), marital status (married/living with a partner, single, divorced/separated/widowed), household income (<$50,000, ≥$50,000 and <$100,000, ≥$100,000), education (less than high school, high school, college/university and higher), employment status (not employed, retired, employed part-time, employed full-time), self-rated health status (very good and excellent, good, fair and poor), family history of cancer (yes/no), personal history of chronic disease (yes/no), and smoking status (current non-smoker/current smoker) obtained from the enrollment questionnaire. PSA and mammography screening testing (yes/no) were used as additional adjustments for men and women, respectively. Papanicolaou (Pap) screening tests were not used in the current analysis since hysterectomy status was not accounted for. The criterion for statistical significance was set at alpha ≤0.05 (2 tailed). All analyses were performed using SAS statistical software (version 9.2 - Linux, SAS Institute, INC., Cary, North Carolina, USA).

## Results

### Sociodemographic characteristics at enrollment

Sociodemographic characteristics of the study participants are summarized in Table 2. At enrollment, most participants lived in an urban setting and with a partner, were well-educated and non-smokers. Greater proportions of men reported higher levels of education, full-time employment, greater household income, living with a partner, being overweight, and personal history of chronic diseases compared to women. The majority of participants fell into the Age-only strata (77%). Participants who reported a bowel condition (PersonalHx and Family/PersonalHx, ~ 10% of participants) were more likely to be current smokers, obese (BMI ≥ 30), and have a personal history of other chronic diseases compared with participants in other strata.

**Table 2:** Characteristics of participants at enrollmen^a^

		Men					Women				
		All men	Stratum				All women	Stratum			
			Age-only	Family Hx	Personal Hx	Family/Personal Hx		Age-only	Family Hx	Personal Hx	Family/Personal Hx
		*n* = 3641	*n* = 2857	*n* = 394	*n* = 316	*n* = 74	*n* = 6000	*n* = 4579	*n* = 810	*n* = 471	*n* = 140
		(37.8%)	78.5%	10.8%	8.7%	2.0%	(62.2%)	76.3%	13.5%	7.9%	2.3%
Age (years; mean ± SD)		56.9 ± 6.1	57.4 ± 5.3	53.8 ± 7.9	55.5 ± 7.5	58.2 ± 7.6	57.0 ± 6.2	57.6 ± 5.4	53.9 ± 8.0	55.7 ± 7.9	58.0 ± 7.0
Residential area	Urban	76.6	76.3	75.1	81.0	78.4	72.8	72.3	74.7	72.8	77.9
	Rural	23.4	23.7	24.9	19.0	21.6	27.2	27.7	25.3	27.2	22.1
Marital status	Married/live with partner	84.5	84.5	84.8	83.2	87.8	74.8	73.9	77.9	76.9	78.6
	Single	5.0	4.5	6.1	7.3	4.1	3.5	3.3	4.7	3.6	3.6
	Divorced/separated/widowed	10.5	11.0	9.1	9.5	8.1	21.7	22.7	17.4	19.5	17.8
Education level	Less than high school	12.0	12.0	13.5	11.1	9.5	11.8	12.2	9.0	13.0	13.6
	High school	13.7	13.8	12.9	15.5	8.1	21.4	20.9	24.2	21.4	19.3
	College/university and higher	74.2	74.1	73.6	73.4	82.4	66.7	66.8	66.8	65.6	67.1
Employment status	Not employed	5.5	5.1	6.6	7.9	4.0	16.7	16.8	15.3	16.6	20.7
	Retired	21.4	21.6	17.3	22.8	33.8	25.1	25.6	20.6	26.5	28.6
	Employed part-time	9.6	9.8	8.6	8.5	12.2	21.4	21.1	24.5	19.3	23.6
	Employed full-time	63.4	63.4	67.5	60.8	50.0	36.7	36.4	39.6	37.6	27.1
Annual household income ($)	< 50,000	27.3	27.1	27.0	29.7	29.7	40.7	41.5	36.1	40.5	41.4
	≥50,000 and < 100,000	43.1	42.8	42.1	45.6	47.3	37.0	37.0	38.5	35.5	32.1
	≥100,000	27.9	28.5	28.4	23.4	21.6	18.8	17.7	23.1	20.4	22.2
BMI (kg/m^2^)^b^	< 18.5	0.1	0.1	0.5	0	0	0.6	0.6	0.7	0.2	0
	≥18.5 and < 25	20.8	21.1	19.3	19.6	23.0	34.4	33.7	40.3	31.4	31.5
	≥25 and < 30	49.8	50.1	50.0	48.4	44.6	37.0	37.4	34.5	36.1	40.7
	≥30	29.0	28.4	29.9	31.7	32.4	27.8	28.1	23.8	32.3	27.1
Smoking status	Current smoker	14.2	13.9	12.9	19.0	16.2	13.6	13.2	13.0	16.1	20.0
	Current non smoker	85.7	86.0	87.1	81.0	83.8	86.3	86.7	86.9	83.7	80.0
Self-reported health status	Excellent/very good	53.7	55.4	55.2	36.2	54.0	56.5	57.2	60.4	44.4	51.4
	Good	38.6	37.4	36.6	51.9	39.2	37.1	36.9	34.6	43.1	40.7
	Fair/poor	7.7	7.2	8.2	11.9	6.8	6.4	5.9	5.0	12.5	7.9
PSA screening	Yes^c^	53.2	53.7	47.2	50.9	74.3	N/A				
Mammography screening	Yes^c^	N/A					93.3	94.9	85.9	90.9	93.6
Family history of cancer	Yes	60.2	54.4	100	53.8	100	64.0	57.2	100	57.3	100
Family history of colorectal cancer	1 FDR^d^ diagnosed > 60 yr	55.5	n/a	56.3	n/a	51.5	58.0	n/a	59.1	n/a	52.0
	1 FDR diagnosed ≤60 yr., or ≥2 FDR	44.5	n/a	43.7	n/a	48.5	42.0	n/a	40.9	n/a	48.0
Personal history of chronic disease^e^	Yes	59.2	59.4	53.8	63.6	59.5	53.6	54.7	45.2	55.0	61.6

^a^Except for age (mean ± SD), values are presented as percentages

^b^Calculated from self-reported height and weight

^c^Ever had a prostate-specific antigen (PSA) or mammography screening

^d^FDR = First-degree relative (mother, father, sister, brother)

^e^Personal history of chronic disease – including angina, chronic bronchitis, cirrhosis of the liver, diabetes, emphysema, heart attack, hepatitis, high blood pressure, high cholesterol, and stroke; excluding bowel conditions (polyps, ulcerative colitis and Crohn’s disease)

Note: A total of 422 participants (132 men, 290 women) had missing data

Stratum Descriptions:

Age-only - participants who should commence screening due to age (50–74 years); considered average-risk

FamilyHx - participants with a first-degree relative diagnosed with CRC; considered moderate-risk

PersonalHx - participants with a personal history of a bowel condition or polyps; considered high-risk

Family/PersonalHx - participants with first-degree relative diagnosed with CRC and personal history of a bowel condition or polyps; considered highest-risk

### Screening status at enrollment and follow-up

Overall, screening status was similar between men and women for all CRC screening tests at both enrollment and follow-up (Table 3). In men, overall CRC screening uptake (including FOBT and/or endoscopy) was low in the average-risk Age-only group at enrollment (26.8% up-to-date, with 57.3% reporting never having had either test), but improved at follow-up (48.3% up-to-date, with 35.8% still reporting never receiving either test). In women, these findings were similar at enrollment (30.3% up-to-date for overall CRC screening, and 51.8% reporting never receiving either test) and follow-up (increased to 51.6% up-to-date for overall CRC screening and “never” reduced to 31.6%). As CRC risk increased across the strata, the proportions of participants who met “up-to-date” status increased, primarily for endoscopy, and the highest proportions of up-to-date status for overall CRC screening were observed in the highest-risk Family/PersonalHx group. A similar trend was observed for FOBT, however the rate of uptake among higher risk participants was much lower than for endoscopic procedures, suggesting that more higher-risk individuals received endoscopy rather than FOBT, consistent with recommendations.

**Table 3:** Colorectal cancer screening status at enrollment and follow-up

		Men - Enrollment				Men - Follow-Up				Women - Enrollment				Women - Follow-Up			
		Age-only	Family Hx	Personal Hx	Family/Personal Hx	Age-only	Family Hx	Personal Hx	Family/Personal Hx	Age-only	Family Hx	Personal Hx	Family/Personal Hx	Age-only	Family Hx	Personal Hx	Family/Personal Hx
Screening Type	Screening Status	%	%	%	%	%	%	%	%	%	%	%	%	%	%	%	%
FOBT	Up to date (< 2 years)	20.3	26.0	24.2	32.1	33.8	30.0	32.4	33.9	21.6	22.3	26.0	35.0	35.1	32.2	29.2	34.6
	Not up to date (≥ 2 years)	14.8	22.5	36.5	28.3	19.0	26.8	39.5	37.3	16.3	25.0	32.1	37.0	22.0	27.8	42.2	41.0
	Never	64.9	51.5	39.3	39.6	47.2	43.2	28.1	28.8	62.1	52.7	41.9	28.0	42.9	40.0	28.6	24.3
Endoscopy^a^	Up to date (< 5 years)^c^	10.2	35.0	68.5	91.9	22.5	58.4	74.2	95.7	13.0	42.5	63.6	88.2	26.1	62.8	71.5	92.9
	Not up to date (≥ 5 years)^c^	7.0	8.7	17.2	4.9	8.3	8.2	17.7	4.3	9.2	10.5	23.7	10.9	9.0	7.2	19.5	7.1
	Never	82.8	56.3	14.3	3.2	69.2	33.4	8.1	0.0	77.8	47.0	12.7	0.8	64.9	30.0	9.0	0.0
Overall CRC^b^	Up to date	26.8	52.8	74.7	95.8	48.3	70.6	85.4	95.7	30.3	54.1	73.1	92.4	51.6	75.5	76.8	94.5
	Not up to date	15.9	15.7	16.2	1.6	15.9	11.3	11.3	4.3	17.9	18.0	19.5	6.7	16.8	10.6	18.9	5.5
	Never	57.3	31.5	9.1	3.2	35.8	18.1	3.3	0.0	51.8	27.9	7.4	0.9	31.6	13.9	4.3	0.0

^a^Endoscopy screening status at follow-up was created by combining colonoscopy and sigmoidoscopy data following recommendations specific to stratum risk

^b^Overall CRC screening status was derived from either FOBT or endoscopy screening status, whichever was more up to date. FOBT, fecal occult blood test; CRC, colorectal cancer screening

^c^At enrollment, 5 years was used as the recommended screening timeframe for endoscopy screening status for all participants, whereas at follow-up, 10 years was used as the recommended timeframe for colonoscopy in Age-only and a subgroup of FamilyHx participants, while 5 years was used for colonoscopy for the remaining FamilyHx participants and the high and highest risk participants, and for sigmoidoscopy for all participants

Stratum Descriptions:

Age-only - participants who should commence screening due to age (50–74 years); considered average-risk

FamilyHx - participants with a first-degree relative diagnosed with CRC; considered moderate-risk

PersonalHx - participants with a personal history of a bowel condition or polyps; considered high-risk

Family/PersonalHx - participants with first-degree relative diagnosed with CRC and personal history of a bowel condition or polyps; considered highest-risk

Among participants who underwent screening at some point (i.e. either up-to-date or not-up-to-date status), approximately 46% of FamilyHx participants with 1 FDR < 60 years or 2 FDR, 59% of PersonalHx and 68% of Family/PersonalHx participants reported a FOBT at enrollment. These proportions were 57, 72 and 74% at follow-up, respectively. For a limited number of participants (for endoscopy and overall CRC screening: 228 and 126 participants, representing 2.36 and 1.31% of the study population, respectively), the same test might have been reported on both surveys and used to determine “up-to-date” screening status, and consequently a regular screening pattern. Overall, these numbers are very low and unlikely to influence the findings reported.

### Screening patterns

**Fig. 1:**
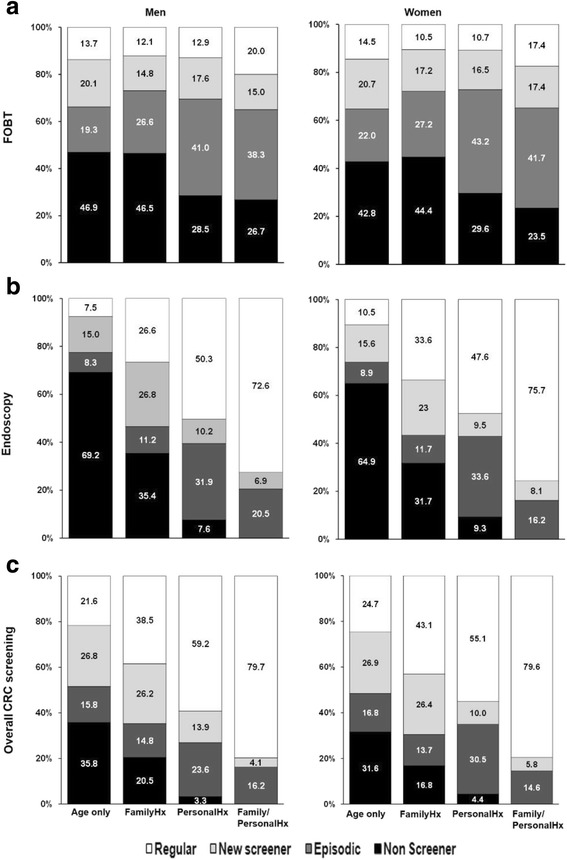
Colorectal cancer (CRC) screening patterns according to four strata of CRC risk. Fecal occult blood test (FOBT; **a**), endoscopy (**b**) and overall CRC screening (**c**). Screening patterns: “non-screeners”, participants who reported “never” being screened at enrollment and follow-up; “episodic” screeners, participants who were “not-up-to-date” or “up-to-date” at enrollment but “not-up-to-date” at follow-up; “new screeners”, participants who were “never” or “not-up-to-date” at enrollment but “up-to-date” at follow-up; and “regular” screeners, participants who reported being “up-to-date” at both enrollment and follow-up. Overall CRC screening based on status for either FOBT or endoscopy. Values on bars are the proportion of participants within each stratum

Patterns of screening behaviour (Fig. 1) showed that for endoscopy and overall CRC screening, the proportions of “non-screeners” decreased, while proportions of “regular screeners” increased as CRC risk increased across strata, in both men and women. Approximately one-third of Age-only participants reported undergoing no screening tests over the course of follow-up, while everyone in the highest-risk Family/PersonalHx group and the majority of the PersonalHx group had at least some screening over the course of follow-up.

### Reasons for screening at follow-up

**Fig. 2:**
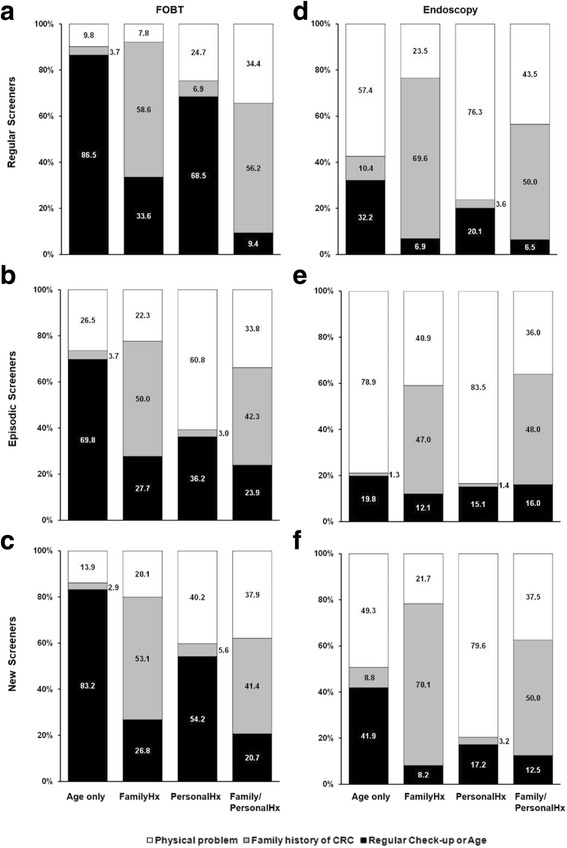
Reasons given for colorectal cancer (CRC) screening tests at follow-up across four CRC risk strata. Fecal occult blood test (FOBT; **a**-**c**) and endoscopy (**d**-**f**) Participants were stratified into four strata of risk nested within different screening patterns, and reasons for screening were reported at follow-up. Options provided included: regular check-up or age, family history of colorectal cancer, and physical problem which included signs of a possible problem or follow-up of previous problem. Participants could select more than one option, but were assigned a primary category based on a priority hierarchy: physical problem, family history and regular check-up or age. Figures **a** & **d** – Regular screeners; Figures **b** & **e** – Episodic screeners; Figures **c** & **f** – New screeners. Values on bars are the proportion of participants within each stratum

Figure 2 describes the primary reasons given for receiving FOBT and endoscopy screening at follow-up, stratified by CRC risk and screening pattern. Average-risk participants (Age-only) indicated a regular check-up or age as the most common reasons for receiving a FOBT, independent of screening pattern (Fig. 2a-c), whereas sign of a physical problem was the more frequent reason for endoscopic procedures (Fig. 2d-f). In moderate-risk participants (FamilyHx), a high proportion of participants indicated family history as the reason for screening for either test type (Fig. 2a-f). However, 40.9% of episodic screeners indicated signs of a physical problem as the reason for endoscopy in this group (Fig. 2e). In participants with a bowel condition (PersonalHx) who were regular and new screeners, regular check-up/age was given as the primary reason for receiving FOBT, whereas the majority reported a physical problem as the primary reason for endoscopy (Fig. 2d-f). Episodic screeners in the PersonalHx group (Fig. 2b) also indicated that a physical problem was the primary reason for FOBT. Reasons for screening in the highest-risk Family/PersonalHx participants were similar between screening tests; the majority of participants reported a family history of CRC or sign of physical problem as reasons for screening (Fig. 2a-f). Compared to regular screeners in the other risk strata, a higher proportion of regular screeners in the Family/PersonalHx group indicated physical problem as a reason for FOBT (Fig. 2a).

### Predictors of screening patterns

**Fig. 3:**
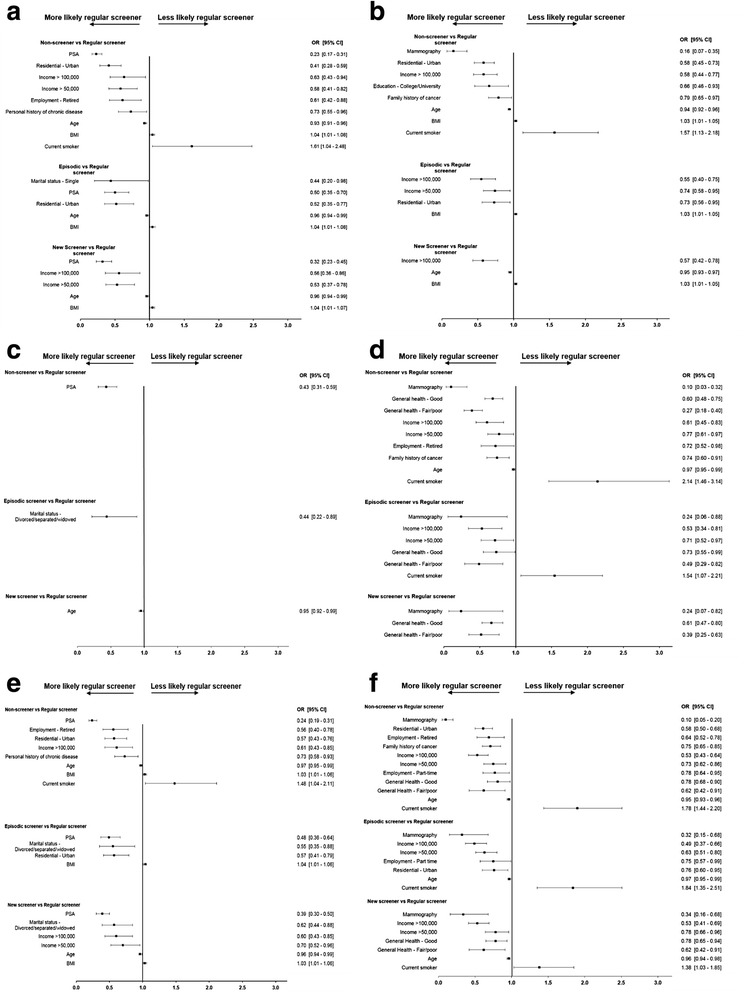
Odds ratios for predictors of colorectal cancer (CRC) screening patterns. Fecal occult blood test (FOBT; **a** – men, **b** - women), endoscopy (**c** – men, **d** - women) and overall CRC (**e** – men, **f** - women) in average-risk participants (Age-only) Data presented as forest plots. Regular screeners were used as the reference group. Variables represent baseline characteristics reported by participants. Only statistically significant predictors are shown

Multinomial logistic regression models were used to determine predictors of screening patterns in average-risk (Age-only) individuals only, due to low sample size in the other three risk strata. Figure 3 illustrates odds ratios for the variables that were associated with FOBT (Fig. 3a-b), endoscopy (Fig. 3c-d) and overall CRC (Fig. 3e-f) screening patterns, using the regular screeners as the reference group.

Overall, across all screening patterns, predictors associated with higher likelihood of being a regular FOBT screener were age, PSA testing (in men, Fig. 3a) and higher levels of household income in women (Fig. 3b), while greater BMI was associated with a marginally lower likelihood in both men (Fig. 3a) and women (Fig. 3b). Compared to non-screeners, male regular screeners for FOBT were more likely to live in an urban location, and report higher income and a history of chronic disease (Fig. 3a). In women, regular FOBT screeners were more likely to be living in an urban location, and report higher education and a family history of cancer compared to non-screeners (Fig. 3b). Current smoking was associated with a greater likelihood of being a non-screener for FOBT in both men and women (Fig. 3a and b). In men, few predictors of regular endoscopy screening were observed (Fig. 3c). In women, lower perception of general health and mammography testing increased the likelihood of regular endoscopy across screening pattern types (Fig. 3d). Higher income, being retired, and family history of cancer were also associated with higher likelihood of being a regular screener compared to a non-screener for endoscopy in women (Fig. 3d). Finally, predictors associated with higher likelihood of regular overall CRC screening across the screening pattern types were PSA testing in men (Fig. 3e) and age, higher levels of household income and mammography in women (Fig. 3f). Current smoking reduced the likelihood of regular overall CRC screening in women (Fig. 3f).

## Discussion

Adherence to a regular screening pattern is the optimal surveillance method to detect potential pre-cancerous lesions or polyps at an early stage, which should lead to earlier detection and reduction of cancer incidence. Here, we investigated screening behaviours over time in a subset of Alberta’s Tomorrow Project participants. The majority fell into the average-risk (Age-only) group, where overall screening uptake was low. However, as the personal risk of CRC increased across strata (from average to highest risk), the uptake of screening and the proportion of participants who were regular screeners also increased. The proportion of average and moderate risk participants up-to-date with screening also increased over time. Reasons for screening were also explored, and, overall across all risk strata, the primary reason for undergoing an endoscopic procedure was sign of a possible problem. Future work should investigate barriers to screening uptake in order to maximize participation.

A previous survey in Alberta reported that 3 years after the release of the Canadian Task Force recommendations in 2001 only 11.9% of average-risk individuals were up-to-date for FOBT screening [29]. Here, we observed that only 21.1 and 34.6% of average-risk participants were up-to-date for FOBT at enrollment and follow-up by 2008, respectively (Table 3). Further, findings from the 2003 Canadian Community Health Survey (CCHS) suggested 85% of average-risk respondents were non-adherent to FOBT recommendations [30], compared with 78.9% in the Age-only group in the current study at enrollment. However, no Albertans were included in the study using 2003 CCHS data. These numbers are well below the goal proposed by the Canadian Partnership Against Cancer of 60% adherence to guaiac or immuno-based fecal tests as the initial recommended test for average-risk individuals [31]. Over the 4.2 year follow-up period, the proportion of participants considered up-to-date for overall CRC screening increased from 28.9 to 50.3% in average-risk (Age-only) ATP participants. Over the same period, approximately 39% of average-risk participants who never had a CRC screening test at enrollment had undergone screening by follow-up. In comparison, an Ontario study reported that approximately 21% of average-risk participants (50–59 years) who never had CRC screening at enrollment completed CRC screening during a 6 year follow-up [32], and a study in an older demographic (65–69 years) in Manitoba reported a significant increase in up-to-date status for CRC screening from 20.5 to 56.9% over a longer follow-up period of 17 years [33]. Compared to international studies, the adherence rate to fecal screening completed over three consecutive years amongst average-risk Australians was 55% [34].

Overall uptake of screening tests increased as risk of CRC increased across the strata. In moderate-risk participants (FamilyHx), 53.7% of participants were up-to-date for overall CRC screening at enrollment and this increased to 73.9% at follow-up. These findings are similar to another Alberta-based study in 2009 investigating first-degree relatives of CRC patients, where 60% of respondents were up-to-date [35]. At both enrollment and follow-up, more participants with a family history of CRC were up-to-date for endoscopy than FOBT, which aligns with the recommendation for individuals with a family history of CRC to undergo endoscopy rather than FOBT. Finally, participants with a bowel condition (PersonalHx) were more likely to be up-to-date for an endoscopy (72.6%) rather than FOBT (30.5%) at follow-up. This is in agreement with a hospital-based Canadian study where 90% of eligible ulcerative colitis patients underwent colonoscopy as part of surveillance screening procedures [36] and supports the observation in the present study that the majority of the PersonalHx participants reported undergoing endoscopy due to a physical problem. Overall, the rate of uptake of screening tests in ATP participants is comparable to other provincial and national cohorts, but there is significant room for improvement as the effectiveness of a screening protocol relies on repeated testing at consistent intervals [25]. Therefore, continued efforts to promote regular screening should be a target for public health initiatives.

Despite the release of the Canadian Task Force screening recommendations in 2001, screening rates remained low among adults 50–74 years in the CCHS conducted 2 years after the recommendations were published [37]. To understand what might be impairing screening participation, a random digit dialing survey of 2500 Canadians aged 50–74 years found most respondents believed CRC screening was important; however, only 40% understood that CRC screening should be done even when asymptomatic [38]. Physicians were reported to have pessimistic expectations on patients’ compliance to CRC screening [39], however, Bryant et al. [38] reported high willingness among patients to talk with a health-care provider about screening. This gap between physician perspectives and expectations on one side, and actual patient attitudes on the other, represents a potential target to increase screening participation and appropriateness.

The 2008 provincial guidelines for CRC screening and the 2001 CTFPHC guidelines were used in the current study since they coincided with the recruitment period of the ATP cohort. Since then, the provincial and CTFPHC guidelines have been updated in 2013 and 2016, respectively [14, 15]. Current provincial guidelines recommend FIT as the initial screening test for average-risk individuals and moderate-risk individuals with one first-degree relative diagnosed ≥60 years [14]. Since November 2013, FIT is available province-wide and the FOBT is no longer in use in Alberta [14]. FIT holds potential for better patient compliance since, unlike FOBT, it does not require dietary or medical restrictions prior to testing [40].

Little information is available on predictors for non-regular cancer screening patterns, such as episodic or new screeners. A study in Australia on participation to four rounds of FIT testing identified those with an “inconsistent participation” pattern (similar to the definition of “episodic” screeners in the present study) to be younger compared to regular participating individuals [41]. These determinants are similar to what we observed here, with older age increasing the likelihood of screening in average-risk (Age-only) participants. Our previous findings within this cohort provided evidence that other cancer screening tests such as PSA and mammography were significant predictors for FOBT uptake [21]. In this prospective analysis, we again show that average-risk participants who continued to undergo FOBT screening on a regular basis were more likely to have other cancer tests completed (Fig. 3). Sewitch et al. [30] reported that “ever use” for CRC screening was associated with older age, higher levels of income, and presence of chronic disease. Similarly, in the 45 and Up cohort, increased uptake of FOBT was associated with higher education and income [42]. In the present study, age, higher income, and adherence to other cancer screening tests were observed to reduce the odds of being a non-screener for FOBT and overall CRC compared to regular screeners in the average-risk participants. Interestingly, family history of cancer was a significant predictor of being a regular screener in women compared to non-screeners, but not men; while in men, personal history of chronic disease was associated with regular screening, indicating regular interactions with a family physician may influence uptake of CRC screening tests [43]. While Australians in the 45 and Up cohort who reported very good or excellent overall health and quality of life were more likely to report having CRC screening, female ATP participants were more likely to report endoscopy if they instead reported good or fair/poor general health. Other health-conscious behaviours such as higher levels of physical activity or higher intakes of fruits and vegetables, are likely to play a role in screening behaviour and should be explored further.

An individual’s CRC risk should be considered in determining the most appropriate screening test; otherwise, overuse and avoidable harm could occur. In particular, colonoscopy has been scrutinized due to health-care costs and system strains, high expertise required to perform the test, and patient level of comfort or willingness [44]. Colonoscopy is recommended for a subgroup of moderate-risk individuals, as well as for high- and highest risk individuals (Table 1); however, approximately 56.5 and 67.2% of those participants reported receiving a FOBT at enrollment and follow-up, respectively. This emphasizes the importance of selecting the most appropriate test in light of the patient’s risk and clinical recommendations and suggests that efforts to prioritize tests based on risk, given the specific criteria laid out in the guidelines, may improve CRC screening program effectiveness [45]. Limited access to colonoscopy providers in some communities may have prevented optimal utilization, causing some moderate-risk or high-risk participants to initially undergo a FOBT, with the possibility of a colonoscopy referral, depending on FOBT results. Ideally, screening date and outcome would have provided valuable information regarding appropriateness of CRC testing in average-risk (Age-only) and moderate-risk (FamilyHx) participants with a family history of CRC in 1 FDR ≥ 60 years. For example, if an average-risk participant had a positive FOBT and was referred for colonoscopy, this would be entirely appropriate and in line with the provincial guidelines. However, neither enrollment nor follow-up questionnaire captured this information, preventing further investigation of CRC screening tests in those participants.

### Strengths & limitations of study

Despite the high costs and long duration required to establish prospective cohort studies, a strength of these study designs is the reduction in recall bias [46], given that self-reported data is commonly used to collect information in such studies. While the accuracy of self-reported data has been scrutinized [47], in a meta-analysis comparing data from self-reported cancer screening to documented medical history of screening tests, FOBT and endoscopy had high sensitivity and specificity [48]. Nonetheless, during enrollment nearly all ATP participants (99%) provided consent to link with administrative data [28]. Thus, future studies could undertake validation of self-reported cancer screening behaviours with medical records. Some participants had moved out of province by follow-up (*n* = 302, 3.2%), which could make the Alberta-specific screening guidelines less applicable; however, given the similarities and overlap in recommendations between the provincial and the CTFPHC (national-based) guidelines, it is unlikely that an out-of-province status would meaningfully influence the findings here, and therefore these participants were included in the current study.

We set a conservative timeframe cut-off of 5 years for defining endoscopy status at enrollment, while we applied the recommended cut-offs for colonoscopy and sigmoidoscopy at follow-up (Table 1). This conservative cut-off may have artificially reduced the number of participants who were up-to-date, while increasing not-up-to-date numbers, for endoscopy at enrollment. However, compared with enrollment, proportions of participants classified as “never” for endoscopy were lower at follow-up, for men and women, which is in agreement with the rest of the findings herein. Provincial guidelines recommend screening to commence 8–10 years after bowel condition onset (applicable to the PersonalHx and Family/PersonalHx strata), as longer duration of IBD is associated with greater CRC risk [18]. However, onset age was not available from the enrollment and follow-up questionnaires administered, and therefore we followed the recommendation that CRC screening should start at a younger age for individuals with a bowel condition and thus screening commencement was set to 40 years in this analysis. Another limitation is that our data lacked the results of screening tests as some patients require active surveillance at a time interval shorter than what is recommended by the provincial and CTFPHC guidelines. While these findings reaffirm previous studies [22, 49], other predictors such as primary care practices (i.e. access to family physician, frequency of routine check-ups) that might influence screening behaviours were not included in this analysis and could be explored in future investigations in this cohort with health-care administration database linkage.

## Conclusion

We observed that ATP participants who are at average-risk for CRC were least adherent to CRC screening recommendations, while those at highest risk were the most adherent. Non-regular screening patterns were most prevalent amongst average- and moderate-risk participants, and represent target groups to promote repeat screening. Low adherence to CRC screening recommendations indicates the need to raise awareness of the most current recommendations – provincial guidelines in 2013 [14] and Canadian Task Force guidelines in 2016 [15] – and promote physician-patient conversations through initiatives at both the health-care system and population level, which has successfully led to higher CRC screening participation rates [50, 51]. Future studies should aim to identify barriers to screening uptake to maximize participation in addition to providing further evidence that regular adherence to screening recommendations reduces the incidence and mortality of CRC.

## Abbreviations

ATP: Alberta’s Tomorrow Project
BMI: Body mass index
CCHS: Canadian Community Health Survey
CI: Confidence interval
CRC: Colorectal cancer
CTFPHC: Canadian Task Force on Preventive Health Care
FDR: First-degree relative
FIT: Fecal immunochemical test
FOBT: Fecal occult blood test
HLQ: Health & Lifestyle Questionnaire
IBD: Inflammatory bowel disease
OR: Odds ratio
PSA: Prostate specific antigen
SD: Standard deviation
